# Fracture Performance of Cementitious Composites Based on Quaternary Blended Cements

**DOI:** 10.3390/ma15176023

**Published:** 2022-08-31

**Authors:** Grzegorz Ludwik Golewski

**Affiliations:** Department of Structural Engineering, Faculty of Civil Engineering and Architecture, Lublin University of Technology, Nadbystrzycka 40 Str., 20-618 Lublin, Poland; g.golewski@pollub.pl; Tel.: +48-81-538-4394; Fax: +48-81-538-4390

**Keywords:** concrete composite, quaternary blended cement (QBC), mineral additives, cracking, fracture toughness, fracture mechanics parameters

## Abstract

This study presents test results and in-depth discussion regarding the measurement of the fracture mechanics parameters of new concrete composites based on quaternary blended cements (QBC). A composition of the two most commonly used mineral additives, i.e., fly ash (FA) and silica fume (SF), in combination with nanosilica (nS), has been proposed as a partial replacement for ordinary Portland cement (OPC) binder. Four series of concrete were made, one of which was the reference concrete (REF) and the remaining three were QBC. During the research, the main mechanical parameters of compressive strength (*f*_cm_) and splitting tensile strength (*f*_ctm_), as well as fracture mechanics parameters and the critical stress intensity factor $\left(K_{\mathrm{Ic}}^{S}\right)$, along with critical crack-tip opening displacements (*CTOD*_c_) were investigated. Based on the tests, it was found that the total addition of siliceous materials, i.e., SF + nS without FA, increases the strength and fracture parameters of concrete by approximately 40%. On the other hand, supplementing the composition of the binder with SF and nS with 5% of FA additive causes an increase in all mechanical parameters by approximately 10%, whereas an increase by another 10% in the FA content in the concrete mix causes a significant decrease in all the analyzed factors by 10%, compared to the composite with the addition of silica modifiers only.

## 1. Introduction

Building structures made of concrete composites effectively protect the occupants against rain, moisture, noise, cold, or temperature fluctuations [1,2]. In addition, the construction of buildings and structures made of concrete composites, as well as the subsequent maintenance and renovation of those concrete structures, catalyzes the development of world economies, thus significantly contributing to their economic progress and an increase in gross domestic products [3]. Therefore, from the economic and social point of view, exploring the dynamics of the development of the concrete industry is still highly desirable [4,5].

Unfortunately, the production process of concrete, which is currently the most utilized basic construction material, is fully against the principles of sustainable development, generating identifiably negative effects on the natural environment. The impact of the lack of ecological production of this very useful construction material concerns mainly the cement matrix of the concrete composite [6,7,8,9,10].

Ordinary Portland cement (OPC), which is the basic binding agent in the production of concrete composites, is formed as a result of burning Portland clinker, which generates significant amounts of harmful greenhouse gases, mainly carbon dioxide (CO_2_), during this process [11,12,13,14]. Securing the aggregate base for the production of over 30 billion tons of concrete per year also entails the impoverishment of the natural environment by another billion tons of aggregates, mainly in mineral form, per annum. If you add to this the fact that the cement production process consumes significant amounts of both thermal and electrical energy [15], then after summing up all the above aspects, concrete in its natural form becomes a definitely non-ecological material [16,17,18,19].

Therefore, in order to reduce the negative environmental impact of only-OPC-based concrete production, measures have been taken to reduce the share of pure Portland clinker in the composition of cement by replacing it with other mineral components in the form of additives [20,21,22,23,24], and recently, also nanoadditives [5,25,26,27,28,29]. Such materials are generally referred to as supplementary cementitious materials (SCMs) [30,31,32,33,34,35,36,37].

It should be noted that the use of multi-component cement containing SCMs will allow scientists to:

- Improve the efficiency of OPC production related to the possibility of using large amounts of mineral additives and nanoadditives, including what is often problematic or even harmful waste [38];
- Achieve a significant reduction in CO_2_ emissions [39];
- Reduce energy consumption [40];
- Meet the United Nations’ sustainable development goals [41,42].

For these reasons, the development of multi-component cements with a diversified composition is justified from an economical and ecological point of view. In addition, the synergistic effect of the interaction of several mineral additives can have a favorable effect on the properties of multi-component cements, compared to cements containing only one mineral additive [43,44,45]. This allows, among others, for preparing concrete composites that are resistant to significant static, impact, and dynamic and fatigue loads [46,47,48,49,50,51,52,53,54,55,56,57,58].

The most frequently used SCMs currently include:

- Fly ash (FA) [59];
- Silica fume (SF) [60];
- Ground or granulated blast-furnace slag (GGBS) [61];
- Nanosilica (nS) [62];
- C-S-H nanoseeds [63,64];
- Waste glass, limestone powder, crumb rubber, and others [65,66].

In addition, synthetic fibers, including polypropylene, basalt, and polyvinyl alcohol (PVA) fibers, can be used in cementitious composites to improve the anti-cracking performance and fracture toughness of the matrix [67,68,69,70].

Unfortunately, a significant factor thus far inhibiting the development of concrete composite production based on multi-component cements is the lack of practical knowledge related to the use of binders of this type in concrete technology. Therefore, this paper presents comprehensive examinations of the fracture mechanics parameters of concrete composites made of quaternary blended cements (QBC).

It should be mentioned that the fracture toughness of concretes containing only FA or SF has been the subject of previous studies. The synergy of the interactions of these two SCMs, in the context of the fracture toughness of concrete, has also been studied. Thanks to previous experiments, for example, the optimal amount of FA in the context of improving fracture resistance and toughness—the critical stress intensity factor ($K_{\mathrm{Ic}}^{S}$) of the fly-ash concrete was established [71]. The influence of the w/c ratio, and its influence regarding SF content on the fracture mechanics parameters $K_{\mathrm{Ic}}^{S}$ and critical crack tip-opening displacement (*CTOD*_c_) of gravel concrete containing SF were investigated in two studies [72,73]. The positive influence of both pozzolanic active mineral additives, i.e., FA + SF, on delays to the cracking processes in ordinary unaged and mature concrete was also identified [60,74,75,76]. Moreover, in other studies [74,75,76], both the previously mentioned parameters of the fracture mechanics, i.e., *K*_Ic_ and *CTOD*_c_, as well as the fracture energy (*G*_F_), were also analyzed.

Previous papers, which presented the results of these and other similar studies, are gathered in Table 1. Table 1 summarizes the previous works related to this topic, with the important findings made in these studies.

However, a new type of cementitious composite in terms of fracture toughness was examined in the current article. The cement matrix in the composites in question was made on the basis of OPC, in connection with two main additives, i.e., FA and SF, and a nanoadditive in the form of nS. In order to track the various components of SCMs, four series of concretes were made, including:

- reference concrete (REF) based only on OPC,
- three composites made of quaternary blended cements (QBC), based on OPC and enhanced by FA, SF, and nS.

The conclusions resulting from the research undertaken—in terms of the synergy of the impact of SCMs on the fracture processes in cementitious composites with a diversified binder composition—may contribute in the future to a more conscious use of such materials in composite structures. Undoubtedly, this will positively affect the reduction of CO_2_ emissions released into the atmosphere, which will be a significant step toward the further development of sustainable construction.

## 2. Experimental Setup

### 2.1. Materials

#### 2.1.1. Aggregates

The following types of aggregates were used in the studies:

- natural gravel, with grain diameters ranging from 2.0 to 8.0 mm and with a specific gravity of 2.65 g/cm^3^—in the form of a coarse aggregate,
- pit sand with a maximum size of 2.0 mm and with a specific gravity of 2.60 g/cm^3^—in the form of a fine aggregate.

#### 2.1.2. Binders

In order to prepare the concrete mixtures, four types of binders were used, including:

- OPC CEM I 32.5 R, produced by the Chełm cement plant,
- Class F FA, produced by Puławy thermal-electric power station,
- non-condensed SF, obtained from Łaziska Ironworks,
- nS Konasil K-200, produced by the OCI Company Ltd (Seoul, Korea).

The chemical compositions of all binders, evaluated using X-ray fluorescence (XRF), are shown in Table 2.

#### 2.1.3. Water

The water used in this experiment is from a domestic water supply that meets the requirements of European Standard EN 1008:2002, i.e., “Mixing water for concrete” [78].

#### 2.1.4. Admixture

A superplasticizer (SP), STACHEMENT 2750, based on polycarboxylates was used in order to improve the flowability of the concrete. The SP accounts for 1.8% of the binder mass, with a very high liquefaction effect that is retained for a longer period than with the more commonly used superplasticizers.

### 2.2. Mix Proportions

Fracture toughness tests to estimate the critical stress intensity factor, $K_{\mathrm{Ic}}^{S}$, and critical crack tip opening displacement, *CTOD*_c,_ as well as the compressive strength, *f*_cm,_ and the splitting tensile strength, *f*_ctm,_ were conducted on four types of concrete composites containing different amounts of the OPC and the additives.

Table 3 presents the mix proportions of concrete composition, with specifications of the percentage contents of individual binders in each concrete mix. In addition, it should be noted that all mixtures had the same water–binder ratio (w/b = 0.4).

### 2.3. Mixing, Casting, and Specimen-Curing

The stages of the mixing procedure of concrete components, including the duration of all of the necessary works, are presented in Table 4.

The total time for preparing the concrete mix is approximately 9–10 min (Table 4). After final preparation, the fresh mixture is poured into molds and compacted on a vibrating table. The concrete cubes are then cast for compressive strength and splitting tensile strength testing, and beams with initial cracks are used for evaluating the fracture toughness parameters, $K_{\mathrm{Ic}}^{S}$ and *CTOD*_c_.

After finishing, the specimens were covered with wet fabric and stored in the casting room at 20 ± 2 °C. In the next step, the specimens were demolded after 48 h and kept in a water tank for the first 14 days. For the next 2 weeks, the specimens were cured under laboratory conditions and then tested 28 days after their preparation.

### 2.4. Methods

#### 2.4.1. Compressive Strength and Splitting Tensile Strength Analysis

During the studies, mechanical property tests were carried out according to the European Standards, i.e.:

- EN 12,390-3:2011 + AC:2012 [79]—in the case of compression strength—*f*_cm_,
- EN 12,390-6:2009 [80]—in the case of splitting tensile strength—*f*_ctm._

Both the mechanical parameters were determined with the use of a Walter + Bai AG hydraulic servo-testing machine, with a maximum bearing capability of 3000 kN and with the application of cubic specimens (150 mm). Six specimens were prepared for each mixture ratio and for both mechanical tests. Therefore, the mean value of the 6 results was taken for the purpose of analysis. During both compression-strength tests and splitting tensile strength tests, the specimens were loaded statically.

#### 2.4.2. Evaluation of Fracture Toughness

**Figure 1:**
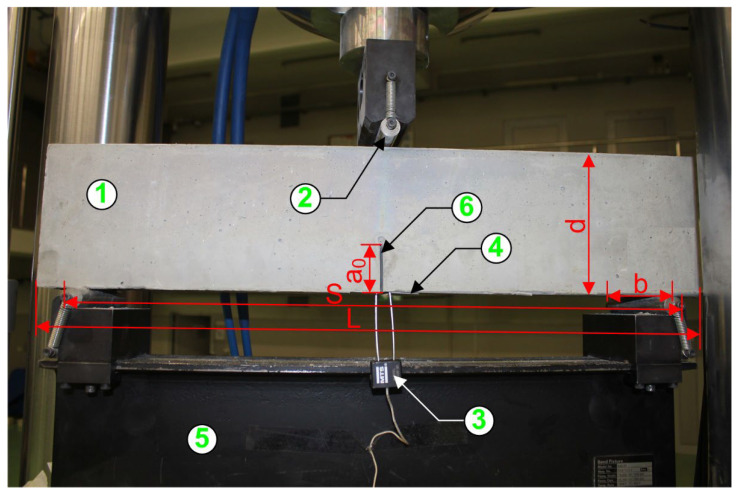
Three-point bending test setup: 1—specimen, 2—load, 3—clip gauge extensometer, 4—clamping test grips, 5—support, 6—initial crack.

The fracture toughness test was carried out in accordance with the RILEM draft recommendations TC-89 FMT [81]: d = 150 mm, b = 80 mm, L = 700 mm, S = 600 mm, a_0_ = 50 mm (Figure 1). A materials test system (MTS) type 810, manufactured by MTS Systems Corp., was used for the fracture toughness tests. Six 80 mm × 150 mm × 700 mm beam specimens with one initial, central crack of 5 mm × 80 mm × 50 mm were prepared for each mixture ratio. The crack opening sensor that was the MTS clip gage axial extensometer 632.03F-3 was used to measure the width of the initial crack opening during the tests. This sensor was placed on the clamping test grips. Figure 1 shows the test setup used for the fracture toughness examinations, with all important details and geometric parameters shown, with a specimen placed under the MTS 810 press.

The analyzed fracture mechanics parameters, i.e., $K_{\mathrm{Ic}}^{S}$, and *CTOD*_c__,_ were determined with the use of the obtained diagrams for load (*F*)—crack mouth opening displacement (*CMOD*); *F*–*CMOD*, and the detailed formulas can be found in [80]. Moreover, two important parameters were needed to determine the fracture toughness factors of the analyzed concretes [81], i.e.:

- maximum force—*F*_max_, loading the specimen,
- a tangent of the *F*–*CMOD* relationship in the first (Ci) and second (Cu) phases.

The critical stress intensity factor $K_{Ic}^{S}$ was calculated from Equation (1) [81]:

(1) $$K_{\mathrm{Ic}}^{S}=3\left(F_{\max}+0.5W\right)\frac{S{\left({\pi a}_{c}\right)}^{1/2}F\left(\alpha \right)}{2W^{2}b}$$

in which:

$$F\left(\alpha \right)=\frac{1.99-\alpha \left(1-\alpha \right)\left(2.15-3.93\alpha +2.7\alpha^{2}\right)}{\sqrt{\pi^{1/2}\left(1+2\alpha \right){\left(1-\alpha \right)}^{3/2}}}$$

where: $\alpha =\frac{a_{c}}{d};\;F_{max}$ is the measured maximum load; *W* $=\frac{W_{0}S}{L};$ $W_{0}$ is the self-weight of the beams d, b, S, and L, as seen in Figure 1.

The second of the analyzed fracture mechanics parameter, i.e., the critical crack tip opening displacement, *CTOD*_c,_ was determined from Equation (2) [81]:

(2) $$CTOD_{c}=\frac{6F_{max}Sa_{c}V_{1}\left(\alpha \right)}{Ed^{2}b}{\left[\left(1-\beta^{2}\right)+\left(1.081-1.149\alpha \right)\left(\beta -\beta^{2}\right)\right]}^{1/2}$$

in which: $\alpha =\frac{a_{c}}{d},\;\beta =\frac{a_{0}}{a_{c}},\;a_{0}$ is the initial notch depth, according to Figure 1.

## 3. Results and Discussion

### 3.1. Mechanical Properties

**Figure 2:**
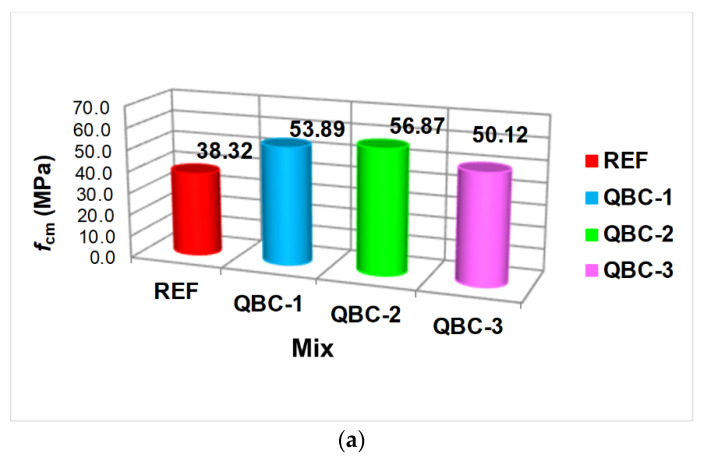

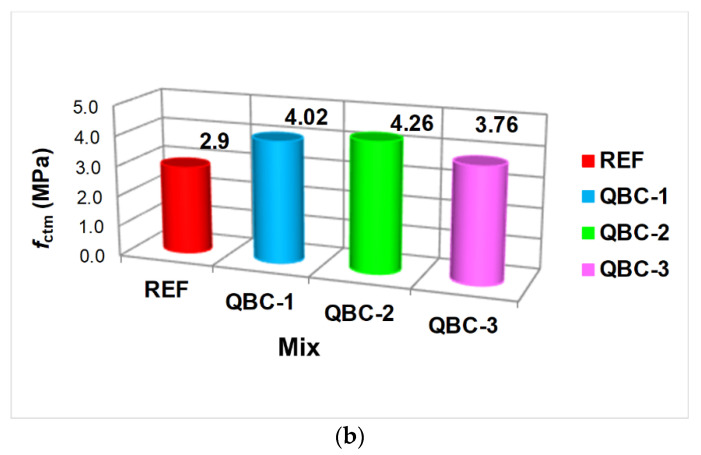
The results of the analyzed mechanical parameters of the concrete composites: (**a**) compressive strength, (**b**) splitting tensile strength.

The results of the tests of the basic strength parameters of concretes with variable structures in terms of the cement matrix are shown in Figure 2. It shows that the proposed material modifications resulted in a very clear improvement in both *f*_cm_ and *f*_ctm_ for all QBCs. Additionally, the upward trends for both analyzed parameters were strictly consistent with each other between the individual materials.

The highest compressive and splitting tensile strengths were obtained for the concrete mixed with the addition of three different SCMs in the composition of the cement matrix (Figure 2). For the QBC-1, QBC-2, and QBC-3, the compressive strengths of concrete composites are 53.89 MPa, 56.87 MPa, and 50.12 MPa, respectively, indicating an increase of 41%, 48%, and 31%, respectively, compared with the working condition of concrete without additives. On the other hand, the corresponding splitting tensile strengths are 4.02 MPa, 4.26 MPa, and 3.76 MPa, respectively, indicating an increase of 39%, 47%, and 30%, respectively, compared with the working condition of the REF group (Figure 2).

**Figure 3:**
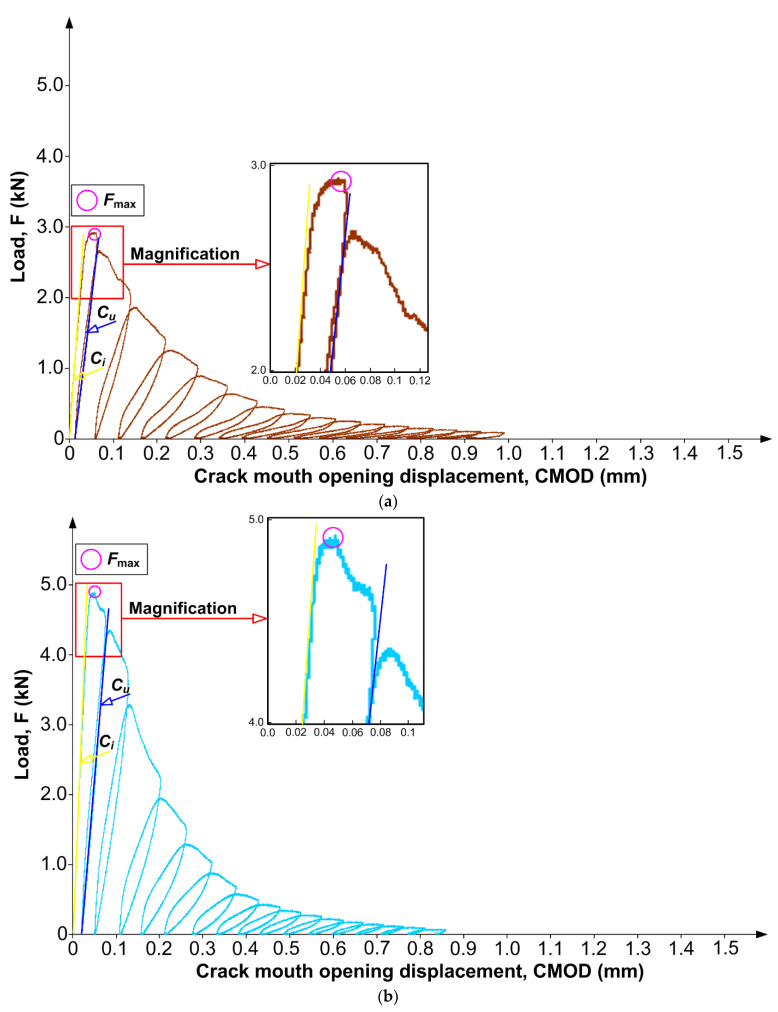

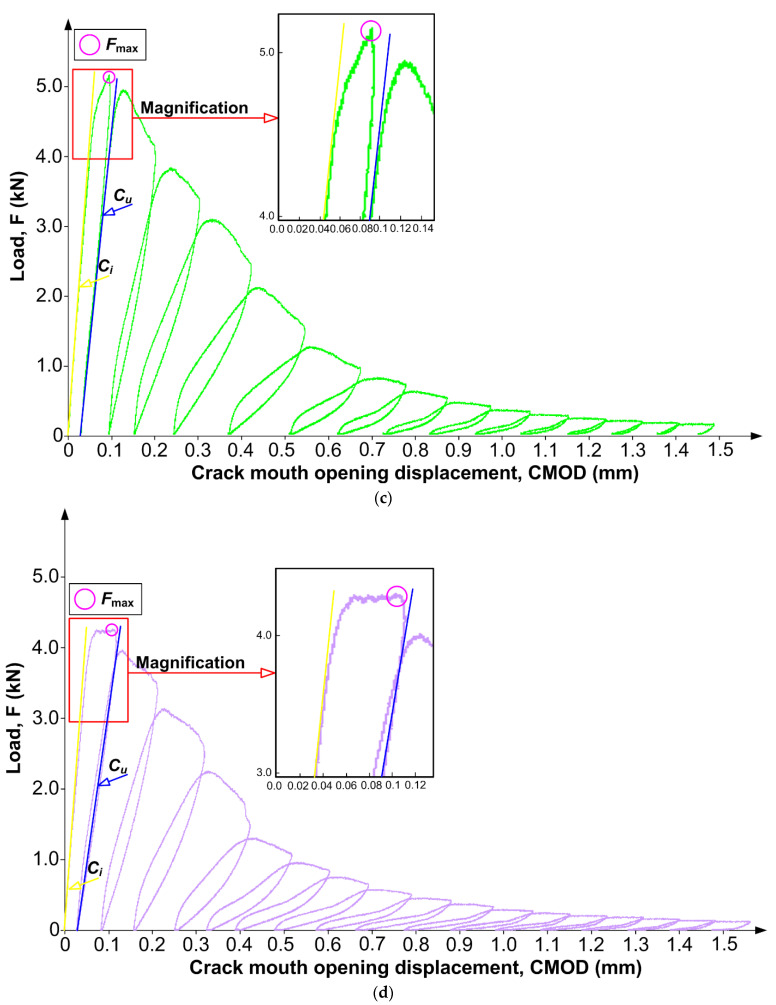
Exemplary load vs. CMOD curves for the analyzed concrete composites: (**a**) REF, (**b**) QBC-1, (**c**) QBC-2, (**d**) QBC-3.

The obtained test results of the mechanical parameters can be explained by the fact that the FA additive, used in small amounts, is able to additionally produce a larger amount of more compact C-S-H phase, which makes the material more airtight by filling the material’s pores [82,83]. However, the presence of FA in the composition of the cement matrix implies a slight reduction in the strength parameters of the composite through the heterogenization of its structure and reduced pozzolanic activity in the initial curing period of materials with these additives [84,85,86]. As a consequence, the smallest effect of improving the composite strength parameters and the increase in heterogeneity in the obtained results were observed in the concrete from series QBC-3 (Figure 2 and Figure 3). The effect appeared, despite the presence of two other, more active, SCMs in the concrete composition—SF and nS.

QBC-1, which contained only two SCMs, showed a much higher value for both *f*_cm_ and *f*_ctm,_ compared to QBC-3. Nevertheless, the results for this material were clearly lower than the values obtained for the matrix-based composite, which was composed of 70% OPC + 5% FA + 10% SF + 5% nS (Figure 2). Therefore, it can be concluded that supplementing the composition of the cement binder with three pozzolanic active ingredients (one of which is FA, in the amount of several percent) causes the occurrence of a strong synergy between all components in the composite structure that clearly increases the material strength indicators (Figure 2). This phenomenon is confirmed by the results of tests on the microstructure of composites of this type, as presented in previously published papers [87,88].

### 3.2. Fracture Toughness

Figure 3 illustrates, separately for each material, the typical exemplary *F–CMOD* curves of the concrete beams after conducted studies. This figure also shows the magnifications of the first two important stages of the beam work during their fracture. In order to better visualize the differences in the fracture processes in individual materials, each of the graphs was made with a different color (Figure 3).

An analysis of the graphs shown in Figure 3 shows that the highest values of the forces *F*_max_, which are clearly exceeding 5.0 kN, were observed for concrete samples from series QBC-2 (Figure 3c). The destruction process in this material showed signs of quasi-plastic fracture. It was shown that the *F–CMOD* curves were inclined, with steadily and slowly declining parts of the plot in the unloading phase and significant increments of *CMOD* intervals between the force *F*_max_ and the moment of specimen destruction (Figure 3c).

The development of damage was visible in a similar way, but with greater intensity, in the concrete with a higher content of FA, i.e., QBC-3 (Figure 3d). As in the case of the composite with an FA content at the level of 5%, a clear slope on the *F–CMOD* graph was also observed in this case, as well as the process of damage development, which took quite a long time until the beams were completely destroyed, with a clear increase in *CMOD* at the subsequent loading stages of the specimens. A significant difference in the concrete of this type, in comparison to QBC-2, was the occurrence of the force *F*_max_ of much lower values, on average, by approximately 1.0 kN (Figure 3d). Therefore, the use of more FA in QBCs resulted in greater continuity of the concrete structure. Unreacted FA grains, giving the composite the characteristics of a quasi-plastic material, are able to extend the destruction process so that composites of this type can accumulate more energy in the process of crack development in the material structure. As a result, it is possible to increase the intervals between the particular phases of crack development, in order to partially slow down this process and, consequently, to significantly extend it. The phenomenon of the non-linear behavior of concretes containing FA is described in detail in [89]. It should be added that similar phenomena of fracture processes can be observed, for example, in concretes containing expanded polystyrene (EPS) [90] or recycled polyethylene terephthalate (PET) [91]. Such an effect was also observed for dispersed reinforcement in the form of basalt fibers at different percentages [92] and in concretes with a 37% volume fraction of coarse aggregate [93].

In the case of the concrete samples from series QBC-1, containing only active SF and nS, the forces of *F*_max_ were only slightly lower than the values obtained for QBC-2 (Figure 3b). The fracture process of this concrete differed significantly, in comparison to the materials with the FA additive. It can be clearly stated that the fracture of beams made from this material was clearly brittle. The *F–CMOD* curve rose sharply to the value *F*_max_ in the first phase of the load; the entire destruction process was much shorter, with a rapidly dropping *F–CMOD*. The *CMOD* increments were also smaller in the subsequent load-unload cycles. The development of damage in the process of the cyclic loading of specimens was, therefore, stable and rapid in this material. Additionally, the structure of QBC with two types of silica materials improves the post-peak compressive behavior of the concrete (Figure 3b). A similar phenomenon in the fracture of concretes with the addition of FA and SF was also observed by the authors of [74,94].

The lowest values of forces *F*_max_, amounting to only 3.0 kN, were recorded during the tests of the fracture processes in the reference concrete (REF). However, the character of the destruction of this composite was similar to the behavior of the quasi-plastic material, although not as evident as in the case of composites with the FA additive (Figure 3a). Additionally, from the magnification of the *F–CMOD* curve, it was possible to observe that it was slightly inclined, with evenly declining sections of the graph in the unloading phase and average increments of *CMOD* intervals between the force *F*_max_ and the moment of specimen destruction (Figure 3a).

**Figure 4:**
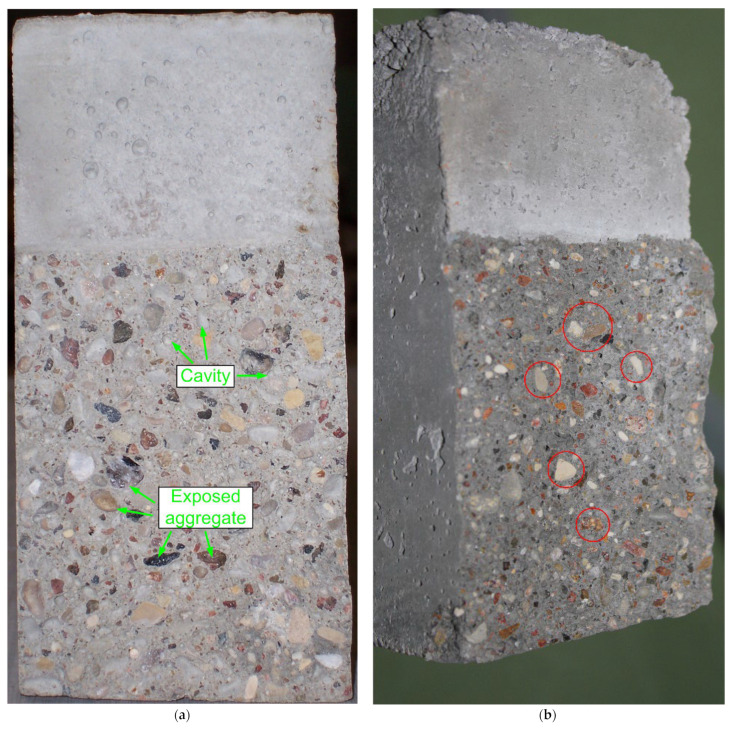

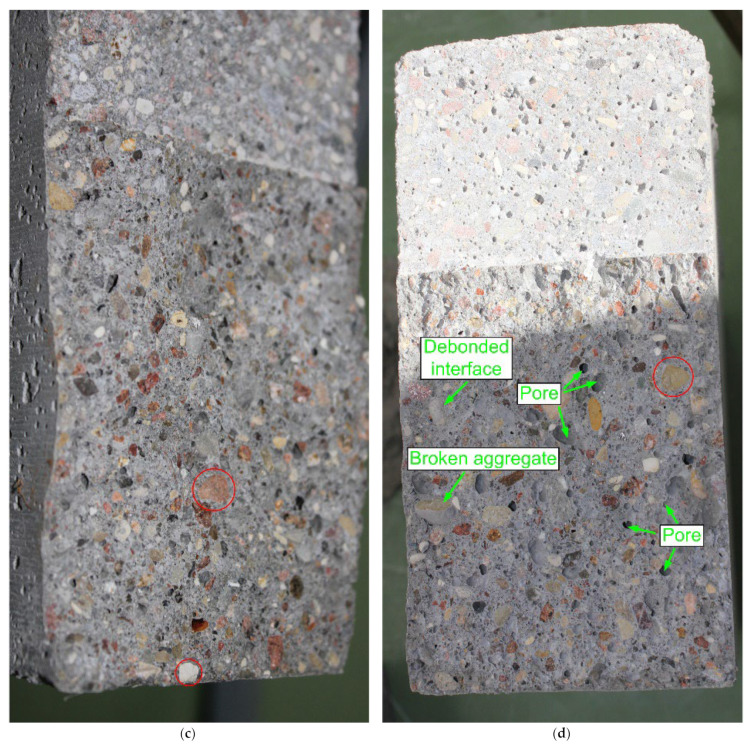
Cross-section of the analyzed concrete composites after a fracture toughness test, with visible characteristic macroscopic structure: (**a**) REF, (**b**) QBC-1, (**c**) QBC-2, (**d**) QBC-3.

In support of the above statements, Figure 4 shows a view of the concrete samples after destruction (macroscopic cross-section) as a result of fracture toughness investigations for all analyzed composites. The characteristic dark color of the matrix in the specimens of the series QBC-1 to QBC-3 resulted from the use of SF binder as one of the modifiers of the composition [73,95].

Based on the analysis of the cross-sections of representative samples, as shown in Figure 4, it can be stated that the results obtained during the fracture toughness tests shown in Figure 3 are clearly consistent with the images of the destruction of individual composites that are visible in macroscopic terms.

The failures of the reference concrete (REF series) samples corresponded to the typical failures for ordinary unmodified concrete. In this concrete, the exposed aggregate grains were visible and its destruction occurred mainly through the failure of the cement matrix. Therefore, besides a great deal of exposed aggregate, cavities in the areas of separation of gravel grains from the weak cement matrix were also observed (Figure 4a).

For concrete from the QBC-1 series (Figure 4b) and partially from the QBC-2 series (Figure 4c), a characteristic type of failure was observed, as seen in brittle materials. Figure 4b and, partially, 4c show numerous broken aggregate grains (marked with red borders in Figure 4). Additionally, the concrete of the QBC-2 series shows the well-compressed structure of the cement matrix. It can be seen that the interfacial transition zone (ITZ) between coarse aggregate and paste in this concrete is compact and there are no clear macrocracks (Figure 4c). This proves that there is a clear synergy between the three pozzolanic materials used in this composite.

On the other hand, concrete with a higher content of FA was characterized by a porous structure, visible damage in the ITZ area, and few broken aggregate grains (Figure 4d). The failure surface of this composite showed an intermediate structure between the reference concrete and the QBC-1 and QBC-2 series concretes (Figure 4).

**Figure 5:**
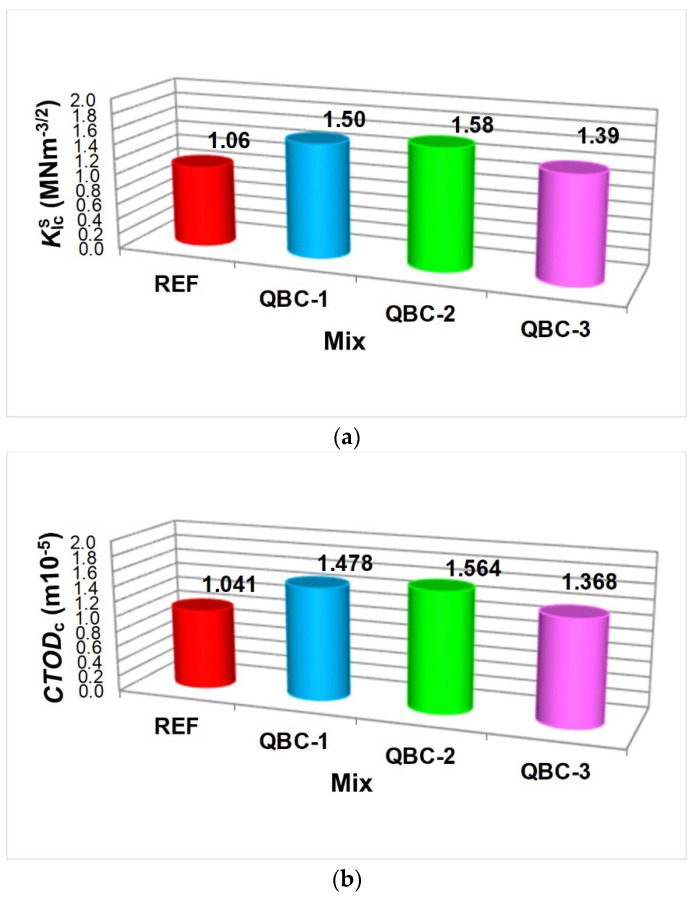
Fracture mechanics parameters results of the analyzed concrete composites: (**a**) $K_{\mathrm{Ic}}^{S}$, (**b**) *CTOD*_c_.

The average values of both parameters of the fracture mechanics obtained in the present experimental tests are presented in Figure 5. As is the case with the results of the strength parameter tests shown in Figure 2, the values of both fracture toughness parameters also show a clear improvement after the use of concrete modification, with the total addition of several SCMs. The QBC-2 had the highest fracture toughness. Both the $K_{\mathrm{Ic}}^{S}$ and *CTOD*_c_ results obtained for this material were approximately 10% higher compared to the values obtained for QBC-1 and approximately 20% higher than for QBC-2. Moreover, the values for each of the analyzed indicators were higher by approximately 50%, compared to those obtained for the reference concrete (Figure 5).

When carefully analyzing the obtained test results of fracture mechanics parameters, it should be stated that for QBC-1, QBC-2, and QBC-3, the $K_{\mathrm{Ic}}^{S}$ of concrete composites are 1.50 MNm^−3/2^, 1.58 MNm^−3/2^, and 1.39 MNm^−3/2^, respectively, indicating an increase of 42%, 49%, and 31%, respectively, compared with the working condition of concrete without additives (Figure 5a). On the other hand, the *CTOD*_c_ are 1.478 m × 10^−5^, 1.564 m × 10^−5^, and 1.368 m × 10^−5^, respectively, indicating an increase of 42%, 50%, and 31%, respectively, compared with the working condition of the REF series (Figure 5b).

## 4. Summary

**Figure 6:**
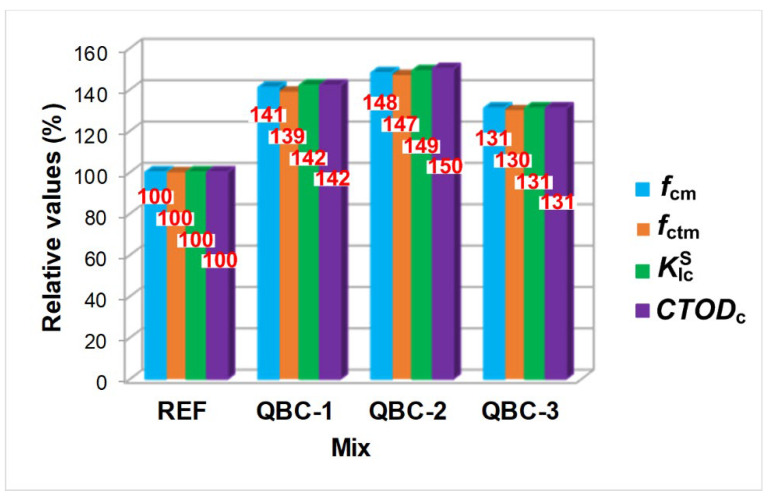
Cumulative comparison of the results obtained for all analyzed parameters and concretes.

On the basis of the first measurements of QBCs fracture mechanics parameters, which contain active substitutes for cement binder in the form of FA, SF, and nS, and analyses of the fracture processes occurring in these composites—under the influence of external loads—Figure 6 summarizes the obtained results of the significant properties of the tested materials. This compilation focuses on the comparison of the relative values of the fracture mechanics parameters, $K_{\mathrm{Ic}}^{S}$ and *CTOD*_c_, as well as the main mechanical parameters, *f*_cm_ and *f*_ctm,_ of the analyzed QBCs.

Based on the graphs, presented in Figure 6, the qualitatively consistent relationship between the fracture mechanics parameters and strength parameters in all analyzed materials can clearly be seen. It shows a very clear convergence in the changes in the values of the analyzed parameters for the individual concretes. An almost perfect results correlation was observed for all the analyzed composites (Figure 6).

In addition, based on the results presented in Figure 3 and Figure 4, Table 5 provides the significant properties of the tested materials, in terms of their fracture toughness. This table presents a comparison of concrete behavior in the process of cyclic loading and unloading, the characteristics of the fracture initiation and propagation process, and the results from the analysis of the fracture surfaces of composites.

## 5. Conclusions

In this paper, the effect of using SCMs with diversified composition as a partial replacement for OPC on the main strength parameters and fracture toughness of concrete during the Mode-I loading of plain concrete was investigated. In the course of the experiments, the composition of the concrete binder was modified with three different materials, including two mineral additives and a nanoadditive. Since the additives, in the form of FA and SF, were used to prepare concrete mixtures, apart from their advantages, they are also problematic industrial wastes (especially FA); therefore, the experiments were also scheduled taking into account the important principles of sustainable development.

It should be emphasized that increasing the share of SCMs in OPC:

- improves the efficiency of OPC production;
- provides a significant reduction in CO_2_ emissions and energy consumption during the production process of OPC;
- meets the demands of ecology;
- is in harmony with the guidelines of sustainable development.

Therefore, based on the conducted studies, the following conclusions can be formulated:

1. The substitution of OPC with the FA + SF + nS combination causes a clear change in the mechanical parameters and fracture toughness of the QBCs.
2. Modification of the binder composition with three pozzolanic active materials resulted in an increase in the analyzed fracture mechanics parameters for each of the combinations, compared to the results obtained for the reference concrete (see Figure 5 and Figure 6) of:
  - approximately −40% for QBC-1,
  - approximately 50% for QBC-2 and
  - approximately 30% for QBC-3.
3. The obtained results for the basic strength parameters, *f*_cm_ and *f*_ctm_, were qualitatively consistent with the results of the fracture mechanics parameters, $K_{\mathrm{Ic}}^{S}$ and *CTOD*_c_ (Figure 6).
4. In the case of concrete from series QBC-1, the development of cracking progressed quite quickly similar like in the completely brittle materials, whereas the addition of FA to the QBC changes the fracture development process in these materials from brittle to quasi-plastic (Figure 3b).
5. The addition of FA to the QBC changes the fracture development process in these materials. The *F*–*CMOD* curves were clearly sloped and the process of fracture development, from the occurrence of *F*_max_ force to the destruction of the specimens, lasted significantly longer than in the case of QBC-1, with a slow increase in strain at subsequent stages of their loading. The energy leading to the final destruction of the specimens was successively accumulated by the material and was used for the development and propagation of intra-material cracks that ran steadily; additionally, it was at a high level of *F*_max_ for QBC-2 (Figure 3c). This phenomenon was determined by the synergistic effect of three pozzolanic, active SCMs, which, in the case of QBC-2, were able to create a compact structure, especially in the area of ITZ responsible for the process of concrete destruction (Figure 4c).
6. As the content of FA rose throughout each of the QBC series, the material became more ductile and showed less brittle failure (Figure 3).

## Figures and Tables

**Table 1:** Papers that presented the results of fracture toughness testing, during Mode-I loading, of concretes containing FA, SF, or FA + SF.

Type of Tested Concrete	Type of Additive Used	Analyzed Fracture Mechanics Parameter	Reference
Plain	FA	$K_{\mathrm{Ic}}^{S}$	[71]
High strength	SF	*K*_Ic_, *CTOD*_c_	[72]
Plain	SF	$K_{\mathrm{Ic}}^{S}$, *CTOD*_c_	[73]
Plain	FA, SF, FA + SF	*G*_F_*, K*_Ic_, *CTOD*_c_	[74]
Plain	FA, FA + SF	*G*_F_*, K*_Ic_, *CTOD*_c_	[75]
Plain	FA, SF, FA + SF	*G*_F_	[76]
High-performance	FA, FA + SF	*G*_F_*, K*_Ic_, *CTOD*_c_	[77]

**Table 2:** Chemical composition of the binders used (mass percentage).

Material\Constituent	SiO_2_	Al_2_O_3_	CaO	MgO	SO_3_	Fe_2_O_3_	K_2_O	P_2_O_5_	TiO_2_	Ag_2_O
OPC	15.00	2.78	71.06	1.38	4.56	2.72	1.21	-	-	-
Class F FA	55.27	26.72	2.35	0.81	0.47	6.66	3.01	1.92	1.89	0.10
Non-condensed SF	91.90	0.71	0.31	1.14	0.45	2.54	1.53	0.63	0.01	0.07
Konasil K-200 nS	>99.8	-	-	-	-	-	-	-	-	-

**Table 3:** Mix proportions (kg/m^3^).

Mix	OPC	%OPC	FA	%FA	SF	%SF	nS	%Ns	Water	SP	Sand	Gravel
REF	352	100	0	0	0	0	0	0	141	0	676	1205
QBC-1	299.2	85	0	0	35.2	10	17.6	5	141	6	676	1205
QBC-2	281.6	80	17.6	5	35.2	10	17.6	5	141	6	676	1205
QBC-3	246.4	70	52.8	15	35.2	10	17.6	5	141	6	676	1205

**Table 4:** Stages of the mixing procedure of concrete components.

Stage Number	Description of the Works	Stage Duration (s)
1	Mix gravel and sand in a drum mixer	120
2	Add the binding materials, i.e., OPC, FA, SF, and mix	180
3	Add the mixture of water, SP, nS, and mix	120
4	Add the remaining water and mix to obtain a homogenous mixture	120–180

**Table 5:** Characteristics features of QBCs, resulting from fracture toughness examinations.

Mix	Analyzed Property		
	The Shape of *F*–*CMOD* Curve	Type of Crack Propagation	Fracture Surface Characteristics
REF	Slightly inclined, with evenly declining sections of the graph in the unloading phase	intermediate fracture between brittle and quasi-plastic	Exposed aggregate, cavities in the areas of separation of gravel grains, weak cement matrix.
QBC-1	Slender, with quickly declining parts of the graph in the unloading phase	clearly brittle fracture	Numerous broken aggregates, compact cement matrix, no clear macrocracks.
QBC-2	Inclined, with steadily and slowly declining parts of the plot in the unloading phase	quasi-plastic fracture	A well-compressed structure of the cement matrix, no clear macrocracks, birthmarks of synergy between the three pozzolanic materials.
QBC-3	Strongly sloped, with very slowly declining sections of the graph in the unloading phase	clearly quasi-plastic fracture	Porous structure, damage in the ITZ area, few broken aggregate grains.

## Data Availability

Not applicable.
